# Amino acid composition of human uterine fluid: association with age, lifestyle and gynaecological pathology

**DOI:** 10.1093/humrep/dev008

**Published:** 2015-02-18

**Authors:** Alexandra J. Kermack, Sarah Finn-Sell, Ying C. Cheong, Nicholas Brook, Judith J. Eckert, Nick S. Macklon, Franchesca D. Houghton

**Affiliations:** 1 Centre for Human Development, Stem Cells & Regeneration, University of Southampton, Southampton SO16 6YD, UK; 2 Academic Unit of Human Development & Health, Faculty of Medicine, University of Southampton, Southampton SO16 6YD, UK; 3 Complete Fertility Centre, Department of Obstetrics & Gynaecology, Princess Anne Hospital, Southampton SO16 6YD, UK; 4 NIHR BRC in Nutrition Southampton, Southampton SO16 6YD, UK

**Keywords:** amino acids, human uterine fluid, BMI, diet, menstrual cycle

## Abstract

**STUDY QUESTION:**

Do the amino acid levels of human uterine fluid vary with age, BMI, phase of menstrual cycle, benign pathology or diet?

**SUMMARY ANSWER:**

The levels of 18 amino acids in human uterine fluid were shown to be affected only by maternal diet.

**WHAT IS KNOWN ALREADY:**

Murine, bovine and ovine uterine amino acid content has been reported, but no reliable data on the human exist. Murine studies have demonstrated that the intrauterine periconceptional nutritional environment is affected by maternal diet.

**STUDY DESIGN, SIZE, DURATION:**

Uterine secretions were aspirated from 56 women aged 18–45 years. The women were recruited preoperatively from gynaecological theatre operating schedules or hysterosalpingo-contrast-sonography (HyCoSy) lists. A proportion of these women had proven fertility; however, the majority were being investigated for subfertility. The BMI, gynaecological history and dietary pattern of these women were also assessed.

**PARTICIPANTS/MATERIALS, SETTING, METHODS:**

Reverse phase high performance liquid chromatography was used to analyse the concentrations of 18 amino acids within the uterine fluid and blood serum. The results were analysed against the women's stage of cycle, age, BMI and diet.

**MAIN RESULTS AND THE ROLE OF CHANCE:**

The profile of 18 amino acids in uterine fluid was described. In total, human uterine fluid was observed to contain an amino acid concentration of 3.54 mM (interquartile range: 2.27–6.24 mM). The relative concentrations of 18 amino acids were not significantly altered by age, BMI, cycle phase or the presence of specific benign gynaecological pathologies. However, a diet identified by a validated scoring system as being less healthy was associated with higher concentrations of asparagine (*P* = 0.018), histidine (*P* = 0.011), serine (*P* = 0.033), glutamine (*P* = 0.049), valine (*P* = 0.025), phenylalanine (*P* = 0.019), isoleucine (*P* = 0.025) and leucine (*P* = 0.043) in the uterine fluid compared with a healthier diet, defined as one with a higher intake of fresh vegetables, fruit, whole-grain products and fish and a low intake of red and processed meat and high fat dairy products. There were no significant correlations between serum amino acid concentrations and those in the uterine fluid.

**LIMITATIONS, REASONS FOR CAUTION:**

Our results enabled us to detect the effect of diet on the concentrations of amino acids in human uterine fluid; however, the study may not have had sufficient numbers to detect mild effects of BMI or age.

**WIDER IMPLICATIONS OF THE FINDINGS:**

These findings increase our understanding of the nutritional environment encountered by the preimplantation embryo, and indicate how periconceptional diet may alter this. Given the importance of early embryo environment for programming of development and future health, this information may aid in the development of nutritional interventions aimed at optimizing the preimplantation phase of human embryo development *in vivo*.

**STUDY FUNDING/COMPETING INTEREST(S):**

This work was funded by the NIHR, the Medical Research Council (G0701153) and the University of Southampton and was supported by the NIHR BRC in Nutrition and Southampton University NHS Foundation Trust. The authors declare no conflicts of interest.

## Introduction

The mammalian embryo has been shown to be able to detect and respond to the intrauterine environment it encounters. A period of developmental plasticity enables the embryo, and later the fetus to alter intrauterine ‘programming’ for later life, as first enunciated by Barker (Barker, 2004). In recent years it has become clear that maternal diet in early pregnancy may also impact on the risk of development of chronic diseases in later life (Barker, 2007); indeed even the environment in which the preimplantation embryo develops has been shown to have significant implications for development and health in later life (Dumoulin *et al.*, 2010; Eskild *et al.*, 2013). Murine studies have demonstrated that a periconceptional low protein diet affects the composition of uterine fluid, and induces a remarkable response in preimplantation embryos, whereby they ‘adapt’ to the low protein environment by increasing endocytosis and trophoblast invasion (Watkins *et al.*, 2008; Eckert *et al.*, 2012; Sun *et al.*, 2014). While uterine fluid represents the preimplantation milieu of the embryo, the nutritional contents of this fluid have not been fully characterized in the human and it remains unclear how these may be altered by factors such as female age, lifestyle and disease. It is recognized that women with an increased age and BMI are more likely to experience subfertility linked to obesity and polycystic ovarian syndrome (PCOS). However, the impact of maternal diet, body composition and age on the metabolic environment of human uterine fluid has not been investigated.

The potential influence of diet on the nutrient composition of uterine secretions has been demonstrated in rats and mice fed a low protein diet during the preimplantation period, showing that diet can shape blastocyst lineage differentiation. In both species there were reduced concentrations of amino acids in the maternal serum (Kwong *et al.*, 2000; Eckert *et al.*, 2012). In rats, this resulted in blastocysts containing a reduced number of inner cell mass (ICM) and trophectoderm (TE) cells which led to abnormal programming of growth (Kwong *et al.*, 2000). In mouse models, a low protein diet fed during the preimplantation period resulted in a reduction in branched chain amino acids in the uterine fluid but in comparison, there were only minimal changes in the amino acid content of the blastocyst (Eckert *et al.*, 2012). These data suggest that, at least in animal models, maternal diet can affect the amino acid environment in which preimplantation embryos develop and may have implications for improving the treatment of infertility, particularly in cases where there is no known cause.

Amino acids have a number of physiological roles during preimplantation development (Houghton, 2013). They may be used as a source of energy (Lane and Gardner, 1998); in the synthesis of proteins and nucleotides (Alexiou and Leese, 1992); as pH regulators (Edwards *et al.*, 1998), antioxidants (Dawson *et al.*, 1998) and osmolytes (Nasr-Esfahani *et al.*, 1992) and as cell signalling molecules (Manser *et al.*, 2004). It has also been demonstrated that amino acids, particularly leucine, have a vital role in the production and regulation of mTOR (mammalian target of rapamycin), a serine/threonine protein kinase which has a crucial role in cell growth and differentiation (Chen *et al.*, 2009).

In contrast to the human, the concentration of amino acids found in the murine (Harris *et al.*, 2005), ovine (Gao *et al.*, 2009) and bovine (Fahning *et al.*, 1967; Shorgan, 2003; Hugentobler *et al.*, 2007) uterine fluids has been described. In the mouse, the uterine fluid contained a total amino acid concentration of 7.18 ± 0.73 mM, which was lower than that observed in the oviduct (Harris *et al.*, 2005). Gao *et al.* examined the changes in amino acid concentrations throughout the menstrual cycle of ewes and found alterations in the levels of asparagine, tyrosine, tryptophan, methionine and valine between Days 3 and 16 of the cycle (Gao *et al.*, 2009). Moreover, an increase in essential amino acids was observed in the uterine fluid of pregnant as opposed to non-pregnant heifers (Groebner *et al.*, 2011). These variations in amino acid concentration with cycle stage and pregnancy suggest that, at least in animal models, levels are regulated.

The inclusion of amino acids in preimplantation embryo culture medium has been shown to be beneficial. In the mouse, non-essential amino acids improve initial cleavage of the embryo whilst the presence of a full complement of amino acids was beneficial for development from the 8-cell to the blastocyst stage (Lane and Gardner, 1997). In the human, embryos cultured in the presence of amino acids produced blastocysts with a greater cell number in both the trophectoderm (TE) and inner cell mass (ICM) (Devreker *et al.*, 2001). Moreover, the ability to use amino acid utilization to predict the future developmental competency of individual human embryos to the blastocyst stage (Houghton *et al.*, 2002; Stokes *et al.*, 2007), as well as to live birth following transfer (Brison *et al.*, 2004), highlights the importance of this nutrient source. These data are intriguing and implicate amino acids as being central for embryo development. It is therefore surprising that the full composition of amino acids in human uterine fluid remains unknown.

In this study we sought to determine the concentration of amino acids in human uterine fluid and how sensitive the amino acid profile is to various factors such as female age, stage of the cycle, reproductive pathology, BMI and diet.

## Materials and Methods

Ethical approval for this study was granted by the Southampton and South West Hampshire Research Ethics Committee (08/H0502/162) and the University Hospital Southampton Research and Development department. A total of 68 women aged 18–45 years were recruited to the study from operating theatre schedules and hysterosalpingo-contrast-sonography (HyCoSy) lists. Exclusion criteria included contraceptive use; current or previous history of malignancy; and known infections (including systemic). Women were recruited preoperatively and gave their written informed consent. Data on the women's demographic, obstetric and gynaecological history, BMI and diet were collected on a standard study proforma on admission. Women taking part in the study were asked about their last menstrual period and length of cycle and from this their stage of cycle was calculated. The women were all ovulating naturally and not undergoing ovarian induction or stimulation. Pathologies were diagnosed by clinical history and examination, laboratory and, ultrasound assessments and when appropriate, laparoscopy. Polycystic ovarian syndrome (PCOS) was diagnosed according to the Rotterdam criteria; leiomyomas and ovarian cysts were diagnosed at ultrasound; and endometriosis and hydrosalpinges were diagnosed at laparoscopy. Diet quality of the women was assessed using a validated food frequency questionnaire (Crozier *et al.*, 2010) to determine their compliance with a ‘prudent’ dietary pattern. This questionnaire has been fully described elsewhere but, briefly, a prudent diet describes a diet characterized by higher intakes of fresh vegetables, fruit, whole-grain products and fish and lower intakes of red and processed meat and high fat dairy products. Depending on the frequency of intake of key dietary elements, a score can be calculated (Crozier *et al.*, 2010). A positive prudent diet score was considered to indicate a ‘healthy’ diet and a negative score to indicate an ‘unhealthy’ diet.

Samples of uterine fluid were obtained after the cervix was cleansed during a speculum examination. This was done prior to commencing the planned surgical or HyCoSy procedure by inserting an embryo transfer catheter (Cook Medical Sydney embryo transfer catheter, USA) gently into the uterine cavity and applying gentle suction with a 2 ml syringe, as previously described (Boomsma *et al.*, 2009). The catheter containing the uterine fluid was placed in a sterile tube and snap frozen in liquid nitrogen, before being stored at −80°C. Sixteen of the women underwent venepuncture, and five of these were fasted prior to the blood test. The amino acid content was determined by reverse phase high pressure liquid chromatography (HPLC).

The uterine fluid samples were removed from the embryo transfer tubing and diluted 1 in 10 in PBS containing 0.1% sodium dodecyl sulphate (Fisher Scientific). Any samples which were heavily blood stained or had a volume <10 μl were discarded. Blood samples were obtained and allowed to clot for 30 min at room temperature. The samples were centrifuged at 2000g for 10 min at 4°C and the serum supernatant collected and stored at −80°C. Serum samples were diluted 1:1 with HPLC grade water prior to analysis. The concentration of amino acids in the uterine fluid and the serum were analysed using reverse phase HPLC (Agilent 1100) and calculated relative to a known concentration of amino acids. Pre-column derivatization was achieved via the automated reaction of 10μl sample and 10 µl *o*-phthaldialdehyde (Sigma) reagent containing 0.2% β2-mercaptoethanol (Sigma). Amino acids were eluted using an elution gradient. Buffer A comprised 15 ml tetrahydrofuran (Fisher Scientific), 200 ml HPLC grade methanol and 800 ml sodium acetate (83 mM, pH 5.9) and buffer B 200 ml sodium acetate (83 mM, pH 5.9; Fisher Scientific) and 800 ml HPLC grade methanol (Christensen *et al.*, 2014). This method allowed the separation and analysis of 18 amino acids; including essential amino acids; histidine (His), glutamine (Gln), arginine (Arg), threonine (Thr), tyrosine (Tyr), methionine (Met), valine (Val), tryptophan (Trp), phenylalanine (Phe), isoleucine (Iso), leucine (Leu), and lysine (Lys); and non-essential amino acids; aspartic acid (Asp), glutamate (Glu), asparagine (Asn), serine (Ser), glycine (Gly), and alanine (Ala). This method did not allow the measurement of proline and cysteine. The amino acid concentration in the uterine fluid was measured for women being treated for subfertility (trying to conceive without success for at least 1 year) (*n* = 51) and those with normal fertility (fertile controls, *n* = 5). The fertile controls were matched as closely as possible to the subfertile group; their average age was 29 years and BMI was 27.5 kg/m^2^.

### Statistical analysis

Statistical analysis was performed using SPSS Statistics 21 (IBM, USA). The data were shown to be not normally distributed using the Anderson–Darling normality test. Results were expressed as an amino acid concentration median ± interquartile range (IQR). Statistical analysis was performed using either Mann–Whitney *U*-test or Kruskal–Wallis test as appropriate; to determine whether the amino acid composition was affected by stage of cycle, age, BMI, prudent diet score or gynaecological history. *P* ≤ 0.05 was considered significant. Spearman's rank correlation was used to examine the relationship between the concentrations of amino acids in the serum and those in the uterine fluid in paired samples.

## Results

### Concentration of amino acids in human uterine fluid

The concentrations of 18 amino acids in 56 human uterine fluid samples were determined using reverse phase HPLC (Table I). The mean age of women participating in the study was 32 years (range 19–45 years); the mean BMI was 25.7 kg/m^2^ (range 17.9–43.6 kg/m^2^). Glutamate was found to be present in the highest concentration followed by glycine and alanine. In contrast, methionine and tryptophan were found to be present in the lowest concentration. In total, human uterine fluid was observed to contain an amino acid concentration of 3.54 mM (IQR: 2.27–6.24 mM).

**Table I:** Concentration of amino acids in human uterine fluid and serum.

Amino acid	Concentration (mM)				
	Uterine fluid median (*n* = 56)	Lower quartile	Upper quartile	Serum mean (*n* = 16)	Spearman's rank correlation
Asp	0.113	0.057	0.199	0.004 ± 0.001	−0.0312
Glu	1.189	0.636	2.059	0.028 ± 0.004	−0.1619
Asn	0.041	0.022	0.062	0.049 ± 0.004	−0.3486
His	0.055	0.029	0.082	0.085 ± 0.006	−0.1047
Ser	0.142	0.072	0.256	0.117 ± 0.009	0.0103
Gln	0.130	0.070	0.223	0.543 ± 0.056	−0.4348
Arg	0.190	0.070	0.314	0.097 ± 0.009	−0.2655
Gly	0.462	0.295	0.953	0.265 ± 0.027	−0.2979
Thr	0.192	0.108	0.310	0.126 ± 0.010	−0.3309
Ala	0.256	0.151	0.530	0.379 ± 0.038	−0.4874
Tyr	0.057	0.040	0.121	0.062 ± 0.006	−0.2322
Met	0.023	0.007	0.049	0.025 ± 0.002	−0.4240
Val	0.114	0.068	0.232	0.193 ± 0.015	−0.1125
Trp	0.043	0.027	0.059	0.074 ± 0.007	−0.4244
Phe	0.048	0.029	0.088	0.059 ± 0.005	−0.3989
Iso	0.047	0.028	0.104	0.061 ± 0.006	−0.3812
Leu	0.093	0.062	0.225	0.112 ± 0.010	−0.2521
Lys	0.209	0.122	0.312	0.213 ± 0.024	0.4760
Total	3.543	2.237	6.271	2.492 ± 0.196	−0.4000
Total essential amino acids	1.188	0.771	2.416	1.650 ± 0.131	−0.2899
Total non-essential amino acids	2.330	1.296	4.150	0.843 ± 0.074	−0.5460

There was no statistically significant difference between the amino acid concentrations found in the uterine fluid of fertile versus subfertile women (*P* = 0.807). Amino acid profiles were also compared between women of proven fertility or who had successfully conceived following assisted reproductive techniques (*n* = 23) and those who did not (*n* = 33). No significant difference was seen between these two groups (*P* = 0.511).

### Female diet alters the concentration of amino acids in the uterine fluid

**Figure 1:**
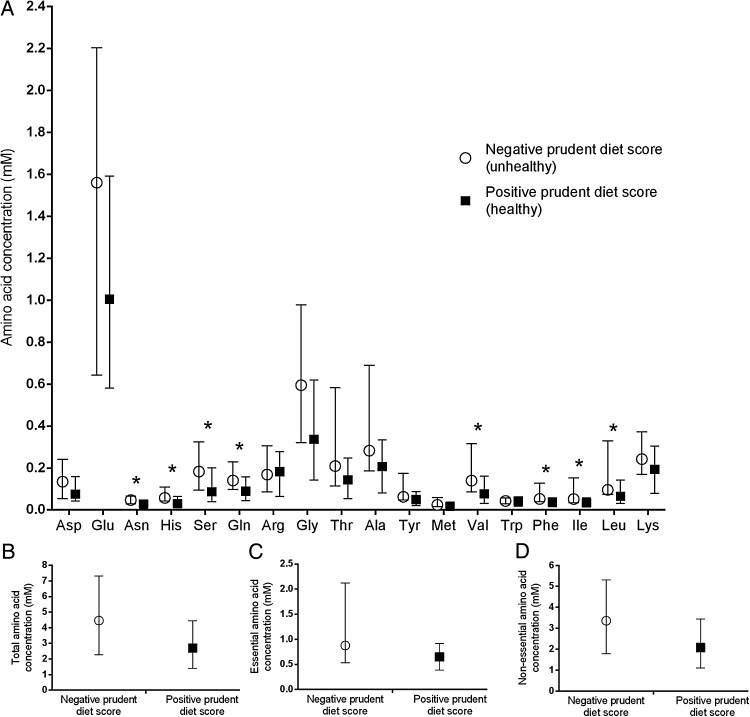
Diet impacts the amino acid composition of uterine fluid in women. The effect of a negative or positive prudent diet score on the (**A**) individual amino acids, (**B**) sum of total amino acids, (**C**) essential and (**D**) non-essential amino acids in the uterine fluid. Values are median ± interquartile range. *n* = 25 for a negative and *n* = 21 for a positive diet score. *P < 0.05.

**Figure 2:**
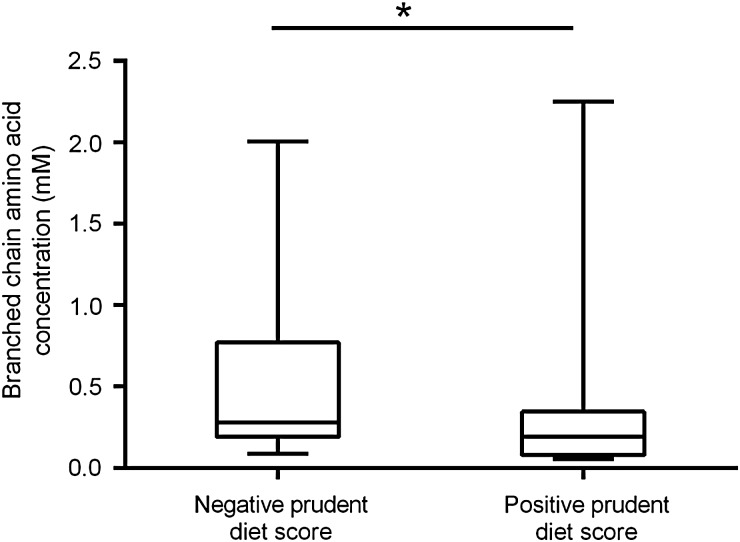
Diet affects the levels of branched chain amino acids in human uterine fluid. Graph demonstrating the difference in concentrations of branched chain amino acids between women with a negative or positive prudent diet score. Values are median ± range. *n* = 25 for a negative and *n* = 21 for a positive diet score. *P < 0.05.

The short diet questionnaire was analysed and participants were given a score based on their answers. In total, 21 females were categorized as having an overall healthy diet while 25 were shown to have an unhealthy diet. The remaining 10 women had missing data meaning it was not possible to calculate a prudent diet score, and these were therefore removed from analysis. There was a significant difference in the uterine fluid concentration of eight amino acids between women with a positive prudent diet score (healthy diet) when compared with those with a negative one (unhealthy diet); asparagine (*P* = 0.018); histidine (*P* = 0.011); serine (*P* = 0.033); glutamine (*P* = 0.049); valine (*P* = 0.025); phenylalanine (*P* = 0.019); isoleucine (*P* = 0.025); and leucine (*P* = 0.043). Significantly higher concentrations of these amino acids were seen in those with an unhealthy diet compared with those who have a healthier diet (Fig. 1). The results also demonstrated a significantly higher concentration of branched chain amino acids in the uterine fluid of women with a negative, compared with a positive, prudent diet score (*P* = 0.030; Fig. 2) but there was no difference in the concentration of either essential or non-essential amino acids between diet types.

### Effect of menstrual cycle stage on the amino acid content of human uterine fluid

**Figure 3:**
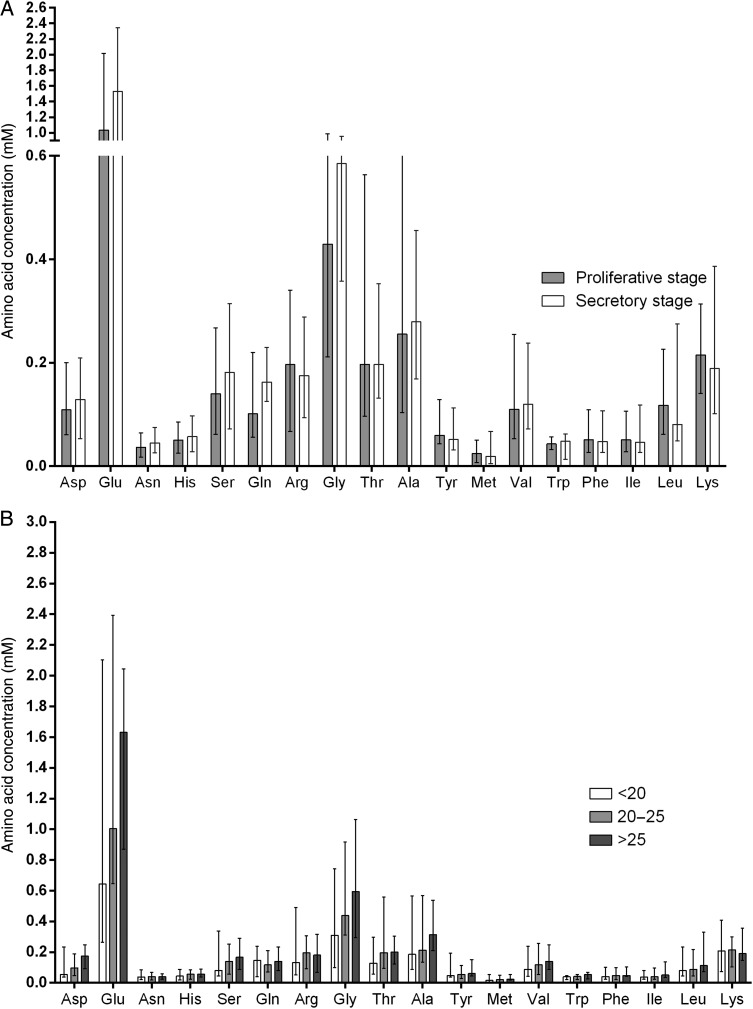
Stage of menstrual cycle and female BMI do not affect the amino acid composition of uterine fluid. Effect of (**A**) menstrual cycle stage and (**B**) female BMI on the amino acid composition of human uterine fluid. Values are median ± interquartile range. (A) For stage of cycle; *n* = 35 for proliferative stage. *n* = 18 for secretory stage. (B) For BMI; *n* = 6 for a BMI <20 kg/m^2^. *n* = 29 for a BMI between 20 and 25 kg/m^2^. *n* = 21 for a BMI over 25 kg/m^2^.

There was no significant difference in the concentration of amino acids in human uterine fluid between the proliferative stage (*n* = 35) and the secretory stage (*n* = 18) of the menstrual cycle (Fig. 3A).

### Effect of BMI on the amino acid content of human uterine fluid

The concentration of amino acids in uterine fluid was measured in women with a BMI of <20 kg/m^2^ (*n* = 6), women with a BMI within the normal range (20–25 kg/m^2^ *n* = 29) and those with a BMI over 25 kg/m^2^ (*n* = 21). Although there was a trend towards increased concentrations of valine, isoleucine and leucine with a rise in BMI, there were no statistically significant differences between the groups (Fig. 3B). This remained true when the groups were corrected for pathology or no pathology, and fertile or subfertile.

### Effect of gynaecological pathology on uterine fluid amino acid content

Study participants were divided into those with no known pathology (*n* = 24) (and either normal fertility or unexplained subfertility) and those with pathology diagnosed either on ultrasound scan or during an operation (*n* = 32). Pathology included submucosal uterine leiomyomas (*n* = 3), ovarian pathology including simple cysts (*n* = 4) or PCOS (*n* = 16), endometriosis (*n* = 3) and hydrosalpinx (*n* = 6). No statistically significant difference in the amino acid concentration in uterine fluid was demonstrated between the groups.

### Effect of female age on the concentration of amino acids in human uterine fluid

The total concentration of amino acids in human uterine fluid in women under 38 years of age (*n* = 47) was not different to that in women older than 38 years (*n* = 9), when a decline in fertility is thought to occur. This remained true when the presence or absence of pathologies were taken into account and when the subfertile group were compared with fertile controls.

### A comparison of the amino acid composition of human serum and uterine fluid

No significant difference was seen between the amino acid concentration of the serum in women who fasted prior to venepuncture and those who did not. The concentration of each amino acid measured was similar in the uterine fluid to that in serum, except for glutamate and aspartic acid which showed more than a 20-fold increase in the uterine fluid when compared with the serum. There was no correlation between the concentration of individual amino acids measured in paired serum and the uterine fluid samples (Table I).

## Discussion

This study reports for the first time the amino acid content of human uterine fluid. It is shown that this is stable through the menstrual cycle, and changes little with increasing reproductive age, BMI or in the presence of a number of benign pathologies. In contrast however, diet is shown to alter amino acid concentration in the uterine fluid and hence, presumably, the nutritional composition within the reproductive tract during preimplantation embryo development.

The techniques used in this study have the advantage that the uterine fluid was collected directly, without the use of lavage. This, together with amino acid measurement using sensitive reverse phase HPLC detection, therefore increases the accuracy of the results obtained. However, a disadvantage of not using uterine flushing was the increased chance of obtaining insufficient sample for analysis.

Studies using a number of animal models suggest that amino acid concentrations in the reproductive tract are actively regulated. The concentration of amino acids found in murine (Harris *et al.*, 2005), ovine (Gao *et al.*, 2009) and bovine (Fahning *et al.*, 1967; Shorgan, 2003; Hugentobler *et al.*, 2007) uterine fluid has been described; however, no one has yet characterized the amino acid profile in human uterine fluid using this methodology. Although the concentrations of amino acids found in human uterine fluid were similar to those observed in the mouse, differences in the levels of aspartate, glutamine, arginine, glycine, alanine were observed, highlighting a variation between species (Harris *et al.*, 2005). In murine studies, amino acid concentration has been found to vary depending on the site within the reproductive tract; higher levels observed in the oviduct when compared with the uterine cavity (Harris *et al.*, 2005). Further studies demonstrated a difference in amino acid concentrations throughout the menstrual cycle in sheep between Days 3 and 16 of the cycle (Gao *et al.*, 2009), and an increase in essential amino acids in pregnancy in bovine research (Groebner *et al.*, 2011). These variations in amino acid concentration with cycle stage and pregnancy suggest that levels in large animal models are regulated, which is in contrast to our observations in the human where no differences were obtained between the proliferative and secretory stages. However, previous work in the human has shown a difference in taurine levels between the mid-cycle and the luteal phase (Casslen, 1987).

Animal studies have also compared the concentration of amino acids found in the Fallopian tube and uterine fluid. In both mice (Harris *et al.*, 2005) and cows (Elhassan *et al.*, 2001), higher concentrations of amino acids were found in the Fallopian tubes. In contrast, the human uterine fluid amino acid concentrations presented in the current work were higher for all amino acids than those reported in the fluid of the human Fallopian tube (Tay *et al.*, 1997). This finding is likely to reflect differences in the techniques used to collect the fluid. In the current study, fluid was obtained *in situ* using an embryo transfer catheter during a HyCoSy investigation or in theatre whereas Tay *et al.* (1997) collected Fallopian tubal fluid following perfusion, which thus may account for their reduced levels. There was no correlation between the concentrations of amino acids observed in uterine fluid and the serum which suggests that the uterus is a protected environment, and is in agreement with work in the mouse (Eckert *et al*., 2012). In both the human and the mouse, glutamate and aspartic acid demonstrated a >20-fold increase in the uterine fluid when compared with the serum.

Although several studies report a decreased clinical pregnancy rate with increased BMI (van der Steeg *et al.*, 2008) and age (Leridon, 2004), a direct effect of diet and lifestyle on the amino acid concentration of uterine fluid has not previously been investigated. Our results suggest that the uterus is a protected environment and the amino acid concentration is not altered by age, BMI or pathology.

Our data show that the amino acid composition of uterine fluid is significantly influenced by a woman's diet; a positive prudent diet score (healthier diet) was associated with a significant reduction in asparagine, histidine, serine, glutamine, valine, phenylalanine, isoleucine and leucine. These findings are comparable to what has previously been observed in mouse models fed a low protein diet, where reductions in the branched chain amino acids were also observed (Eckert *et al.*, 2012). This suggests that, like the mouse, the nutritional environment of human uterine fluid is sensitive to female diet.

This study has not investigated whether a higher or lower amino acid concentration in human uterine fluid improves conception rates, or whether it is the homeostasis of the environment that is essential. However, the quiet embryo hypothesis suggests that a low amino acid turnover improves embryo development (Houghton *et al.*, 2002; Leese, 2002). Recently it has been shown that the decidualised endometrium acts as a biosensor of embryo quality (Brosens *et al.*, 2014). It could therefore be proposed that women with a more prudent diet may promote a low amino acid environment *in utero* which selectively supports the development of high quality, metabolically quiet embryos. This is of particular relevance as in this study, serine and leucine showed a statistically significant reduction in the uterine fluid of women with a positive prudent diet score when compared with those with a negative score, and these amino acids have previously been demonstrated as predictors of embryo viability (Brison *et al.*, 2004).

These data offer the potential to facilitate the production of embryo culture media containing physiologically relevant concentrations of amino acids based on those found in uterine fluid, and perhaps also to guide preconception dietary interventions to optimize the intrauterine environment. In clinical practice, IVF laboratories use embryo culture media whereby the nutritional content has been extrapolated from data obtained from murine embryo development (Gardner and Lane, 1998) and thus may not reflect the nutritive requirement of the developing human embryo as it moves through the reproductive tract (Houghton, 2012). Given that there is now significant, albeit controversial (Carrasco *et al.*, 2013; Lin *et al.*, 2013), evidence supporting the profound influence of the preimplantation environment on subsequent birthweight (Dumoulin *et al.*, 2010; Eskild *et al.*, 2013), it is possible that the inclusion of physiological concentrations of pleiotropic nutrients, such as amino acids, could further enhance the success of clinical IVF.

In conclusion, these data provide the first evidence to suggest that differences in women's diet quality can alter the amino acid concentration of human uterine fluid. Further research is required to examine the impact of the human periconception diet on both the uterine environment and embryo development.

## Authors' roles

Y.C.C., J.J.E., N.S.M. and F.D.H. conceived the experiments. A.J.K., Y.C.C., N.S.M., J.J.E. and F.D.H. designed the experiments. A.J.K. and S.F.-S. performed the experiments. N.B. and Y.C.C. collected samples. A.J.K. and S.F.-S. analysed the data. All authors were involved in the preparation of the manuscript.

## Funding

This work was funded by the NIHR, the Medical Research Council (G0701153), Infertility Research Trust and the University of Southampton. This report is independent research by the National Institute for Health Research Biomedical Research Centre Funding Scheme. The views expressed in this publication are those of the author(s) and not necessarily those of the NHS, the National Institute for Health Research or the Department of Health. Funding to pay the Open Access publication charges for this article was provided by the MRC UK.

## Conflict of interest

None declared.

## References

[DEV008C1] Alexiou M, Leese HJ (1992). Purine utilisation, *de novo* synthesis and degradation in mouse preimplantation embryos. Development.

[DEV008C2] Barker DJ (2004). Developmental origins of adult health and disease. J Epidemiol Community Health.

[DEV008C3] Barker DJ (2007). The origins of the developmental origins theory. J Intern Med.

[DEV008C4] Boomsma CM, Kavelaars A, Eijkemans MJ, Amarouchi K, Teklenburg G, Gutknecht D, Fauser BJ, Heijnen CJ, Macklon NS (2009). Cytokine profiling in endometrial secretions: a non-invasive window on endometrial receptivity. Reprod Biomed Online.

[DEV008C5] Brison DR, Houghton FD, Falconer D, Roberts SA, Hawkhead J, Humpherson PG, Lieberman BA, Leese HJ (2004). Identification of viable embryos in IVF by non-invasive measurement of amino acid turnover. Hum Reprod.

[DEV008C6] Brosens JJ, Salker MS, Teklenburg G, Nautiyal J, Salter S, Lucas ES, Steel JH, Christian M, Chan YW, Boomsma CM (2014). Uterine selection of human embryos at implantation. Sci Rep.

[DEV008C7] Carrasco B, Boada M, Rodriguez I, Coroleu B, Barri PN, Veiga A (2013). Does culture medium influence offspring birth weight?. Fertil Steril.

[DEV008C8] Casslen BG (1987). Free amino acids in human uterine fluid. Possible role of high taurine concentration. J Reprod Med.

[DEV008C9] Chen X, He J, Ding Y, Zeng L, Gao R, Cheng S, Liu X, Wang Y (2009). The role of MTOR in mouse uterus during embryo implantation. Reproduction.

[DEV008C10] Christensen DR, Calder PC, Houghton FD (2014). Effect of oxygen tension on the amino Acid utilisation of human embryonic stem cells. Cell Physiol Biochem.

[DEV008C11] Crozier SR, Inskip HM, Barker ME, Lawrence WT, Cooper C, Robinson SM (2010). Development of a 20-item food frequency questionnaire to assess a ‘prudent’ dietary pattern among young women in Southampton. Eur J Clin Nutr.

[DEV008C12] Dawson KM, Collins JL, Baltz JM (1998). Osmolarity-dependent glycine accumulation indicates a role for glycine as an organic osmolyte in early preimplantation mouse embryos. Biol Reprod.

[DEV008C13] Devreker F, Hardy K, Van den Bergh M, Vannin AS, Emiliani S, Englert Y (2001). Amino acids promote human blastocyst development *in vitro*. Hum Reprod.

[DEV008C14] Dumoulin JC, Land JA, Van Montfoort AP, Nelissen EC, Coonen E, Derhaag JG, Schreurs IL, Dunselman GA, Kester AD, Geraedts JP (2010). Effect of *in vitro* culture of human embryos on birthweight of newborns. Hum Reprod.

[DEV008C15] Eckert JJ, Porter R, Watkins AJ, Burt E, Brooks S, Leese HJ, Humpherson PG, Cameron IT, Fleming TP (2012). Metabolic induction and early responses of mouse blastocyst developmental programming following maternal low protein diet affecting life-long health. PLoS One.

[DEV008C16] Edwards LJ, Williams DA, Gardner DK (1998). Intracellular pH of the mouse preimplantation embryo: amino acids act as buffers of intracellular pH. Hum Reprod.

[DEV008C17] Elhassan YM, Wu G, Leanez AC, Tasca RJ, Watson AJ, Westhusin ME (2001). Amino acid concentrations in fluids from the bovine oviduct and uterus and in KSOM-based culture media. Theriogenology.

[DEV008C18] Eskild A, Monkerud L, Tanbo T (2013). Birthweight and placental weight; do changes in culture media used for IVF matter? Comparisons with spontaneous pregnancies in the corresponding time periods. Hum Reprod.

[DEV008C19] Fahning ML, Schultz RH, Graham EF (1967). The free amino acid content of uterine fluids and blood serum in the cow. J Reprod Fertil.

[DEV008C20] Gao H, Wu G, Spencer TE, Johnson GA, Li X, Bazer FW (2009). Select nutrients in the ovine uterine lumen. I. Amino acids, glucose, and ions in uterine lumenal flushings of cyclic and pregnant ewes. Biol Reprod.

[DEV008C21] Gardner DK, Lane M (1998). Culture of viable human blastocysts in defined sequential serum-free media. Hum Reprod.

[DEV008C22] Groebner AE, Rubio-Aliaga I, Schulke K, Reichenbach HD, Daniel H, Wolf E, Meyer HH, Ulbrich SE (2011). Increase of essential amino acids in the bovine uterine lumen during preimplantation development. Reproduction.

[DEV008C23] Harris SE, Gopichandran N, Picton HM, Leese HJ, Orsi NM (2005). Nutrient concentrations in murine follicular fluid and the female reproductive tract. Theriogenology.

[DEV008C24] Houghton FD (2012). Media composition: amino acids and cellular homeostasis. Methods Mol Biol.

[DEV008C25] Houghton F, Gardner DK, Sakkas D, Seli E, Wells D (2013). Identification of viable embryos by noninvasive measurement of amino acids in culture media. Human Gametes and Preimplantation Embryos.

[DEV008C26] Houghton FD, Hawkhead JA, Humpherson PG, Hogg JE, Balen AH, Rutherford AJ, Leese HJ (2002). Non-invasive amino acid turnover predicts human embryo developmental capacity. Hum Reprod.

[DEV008C27] Hugentobler SA, Diskin MG, Leese HJ, Humpherson PG, Watson T, Sreenan JM, Morris DG (2007). Amino acids in oviduct and uterine fluid and blood plasma during the estrous cycle in the bovine. Mol Reprod Dev.

[DEV008C28] Kwong WY, Wild AE, Roberts P, Willis AC, Fleming TP (2000). Maternal undernutrition during the preimplantation period of rat development causes blastocyst abnormalities and programming of postnatal hypertension. Development.

[DEV008C29] Lane M, Gardner DK (1997). Differential regulation of mouse embryo development and viability by amino acids. J Reprod Fertil.

[DEV008C30] Lane M, Gardner DK (1998). Amino acids and vitamins prevent culture-induced metabolic perturbations and associated loss of viability of mouse blastocysts. Hum Reprod.

[DEV008C31] Leese HJ (2002). Quiet please, do not disturb: a hypothesis of embryo metabolism and viability. Bioessays.

[DEV008C32] Leridon H (2004). Can assisted reproduction technology compensate for the natural decline in fertility with age? A model assessment. Hum Reprod.

[DEV008C33] Lin S, Li M, Lian Y, Chen L, Liu P (2013). No effect of embryo culture media on birthweight and length of newborns. Hum Reprod.

[DEV008C34] Manser RC, Leese HJ, Houghton FD (2004). Effect of inhibiting nitric oxide production on mouse preimplantation embryo development and metabolism. Biol Reprod.

[DEV008C35] Nasr-Esfahani MH, Winston NJ, Johnson MH (1992). Effects of glucose, glutamine, ethylenediaminetetraacetic acid and oxygen tension on the concentration of reactive oxygen species and on development of the mouse preimplantation embryo *in vitro*. J Reprod Fertil.

[DEV008C36] Shorgan LRWLWSB (2003). Free amino acid content of oviductal and uterine fluid at different oestrous stages in the cow. Curr Zool.

[DEV008C37] Stokes PJ, Hawkhead JA, Fawthrop RK, Picton HM, Sharma V, Leese HJ, Houghton FD (2007). Metabolism of human embryos following cryopreservation: implications for the safety and selection of embryos for transfer in clinical IVF. Hum Reprod.

[DEV008C38] Sun C, Velazquez MA, Marfy-Smith S, Sheth B, Cox A, Johnston DA, Smyth N, Fleming TP (2014). Mouse early extra-embryonic lineages activate compensatory endocytosis in response to poor maternal nutrition. Development.

[DEV008C39] Tay JI, Rutherford AJ, Killick SR, Maguiness SD, Partridge RJ, Leese HJ (1997). Human tubal fluid: production, nutrient composition and response to adrenergic agents. Hum Reprod.

[DEV008C40] van der Steeg JW, Steures P, Eijkemans MJC, Habbema JDF, Hompes PGA, Burggraaff JM, Oosterhuis GJE, Bossuyt PMM, van der Veen F, Mol BWJ (2008). Obesity affects spontaneous pregnancy chances in subfertile, ovulatory women. Hum Reprod.

[DEV008C41] Watkins AJ, Ursell E, Panton R, Papenbrock T, Hollis L, Cunningham C, Wilkins A, Perry VH, Sheth B, Kwong WY (2008). Adaptive responses by mouse early embryos to maternal diet protect fetal growth but predispose to adult onset disease. Biol Reprod.

